# Modulating functional and dysfunctional mentalizing by transcranial magnetic stimulation

**DOI:** 10.3389/fpsyg.2014.01309

**Published:** 2014-11-18

**Authors:** Tobias Schuwerk, Berthold Langguth, Monika Sommer

**Affiliations:** ^1^Department of Psychology, Ludwig-Maximilians-UniversityMunich, Germany; ^2^Department of Psychiatry and Psychotherapy, University of RegensburgRegensburg, Germany

**Keywords:** transcranial magnetic stimulation, social cognition, mentalizing, theory of mind, autism spectrum disorders, major depressive disorder, psychiatric disorders

## Abstract

Mentalizing, the ability to attribute mental states to others and oneself, is a cognitive function with high relevance for social interactions. Recent neuroscientific research has increasingly contributed to attempts to decompose this complex social cognitive function into constituting neurocognitive building blocks. Additionally, clinical research that focuses on social cognition to find links between impaired social functioning and neurophysiological deviations has accumulated evidence that mentalizing is affected in most psychiatric disorders. Recently, both lines of research have started to employ transcranial magnetic stimulation: the first to modulate mentalizing in order to specify its neurocognitive components, the latter to treat impaired mentalizing in clinical conditions. This review integrates findings of these two different approaches to draw a more detailed picture of the neurocognitive basis of mentalizing and its deviations in psychiatric disorders. Moreover, we evaluate the effectiveness of hitherto employed stimulation techniques and protocols, paradigms and outcome measures. Based on this overview we highlight new directions for future research on the neurocognitive basis of functional and dysfunctional social cognition.

## Introduction

In the middle of the night your neighbor is desperately trying to open your door with his key. Why is he doing that and how will you react? Knowing that he just came back from a birthday party and being aware of his drinking habits, you are able to infer that he probably falsely believes it is his door he is trying to open. Instead of calling the police you might then help him to find his own apartment. This example illustrates how our ability to understand other people's behavior by attributing mental states like beliefs, desires or intentions, also known as Theory of Mind (ToM) reasoning or mentalizing, drives social interactions.

A fast growing body of evidence suggests that mentalizing is affected in most psychiatric disorders, including but not limited to major depressive disorder (MDD; see Schreiter et al., 2013), bipolar disorder (Bora et al., 2005; Van Rheenen and Rossell, 2013), social anxiety (Ribeiro and Fearon, 2010; Samson et al., 2012), borderline personality disorder (Ghiassi et al., 2010; Mier et al., 2013), eating disorders (Schulte-Rüther et al., 2012) and neurodegenerative diseases (Le Bouc et al., 2012; Poletti et al., 2012). Moreover, it has long been hypothesized that social cognitive deficits in autism spectrum disorders (ASD) and schizophrenia result from impaired mentalizing (Brüne and Brüne-Cohrs, 2006; Frith, 2012).

However, this research is still in an early stage. Inconclusive findings (e.g., Arntz et al., 2009; Schreiter et al., 2013) and a heterogeneous conceptualization of impaired social cognition do not yet allow for drawing firm conclusions about the role of impaired mentalizing in psychiatric disorders. To resolve this ambiguity, it has been suggested to focus on the neurocognitive building blocks of mentalizing in order to find links between symptomatic impairment of social interactions and neurophysiological deviations in psychiatric disorders (Frith, 2012; Kennedy and Adolphs, 2012; Happé and Frith, 2014).

Recently, cognitive neuroscientists started to specify these neurocognitive building blocks of mentalizing using transcranial magnetic stimulation (TMS, Hetu et al., 2012). A coil, placed on the skull over the brain area of interest, produces a focal magnetic field which passes through the skull largely undistorted and induces neuronal depolarization in superficial cortical areas. When TMS is applied over specific brain regions in the context of a cognitive task, the interference with behavioral performance enables the study of causal relations between brain activity, cognitive processes, and behavior (Walsh and Cowey, 2000; Robertson et al., 2003; Paus, 2005).

TMS studies on mentalizing almost exclusively use repetitive TMS (rTMS), which is why the current review focuses on this method. A detailed description of recent technical and methodological issues for the application of various TMS protocols in the study of cognition is provided elsewhere (Sandrini et al., 2011). Applying rTMS has both an immediate interrupting effect on neuronal processing in the stimulated area, and a modulatory after-effect, which outlasts the stimulation period by minutes to hours (cf., Eisenegger et al., 2008). The direction of this after-effect (inhibitory/excitatory) depends on stimulation parameters and baseline activity of the stimulated area. Accordingly, rTMS can be applied during the performance of a cognitive task (online) or before task performance (offline). A third possibility is the application of single pulses which interrupt neuronal activity for a short but well-defined period, useful for identifying temporal characteristics of neurocognitive processes.

Parallel to TMS research on functional mentalizing, clinical research began to employ TMS to treat impaired mentalizing in psychiatric disorders. For this purpose, repeated sessions of rTMS have been applied over periods of several weeks for the treatment of mentalizing deficits in ASD and MDD (Enticott et al., 2011, 2014; Berlim et al., 2012).

Here, we integrate and evaluate findings of these separately emerging lines of research to show how TMS can advance our understanding of the neurocognitive basis of mentalizing and its impairment in clinical conditions, specifically in ASD and MDD. Further, Table 1 provides methodological details of hitherto available brain stimulation studies on mentalizing. We suggest that TMS combined with sensitive experimental paradigms is a promising method to specify the neurocognitive architecture of functional and dysfunctional mentalizing, which can be the key to elucidate impaired social functioning in psychiatric disorders.

**Table 1:** **Methodological details and key findings of available TMS studies on mentalizing sorted by targeted brain region**.

**Study**	**Brain region**	**Technique**	**Targeting procedure**	**Stimulation and Control**	**Paradigm**	**Outcome measure**	Effect size^*^	**Key findings**
**RTPJ**								
Costa et al., 2008	LTPJ; RTPJ	rTMS	Neuronavigation to a priori defined coordinates	1 Hz, 15 min, 90% RMT; sham	Short stories: false belief/faux pas/control	PCR; RT	n/a	rTMS over RTPJ impaired performance in false belief task and in faux pas test
Giardina et al., 2011	LTPJ; RTPJ	rTMS	Neuronavigation to a priori defined coordinates	1 Hz, 600 stimuli, 90% RMT; control (10/20 EEG position Oz)	Social interaction scenarios requiring either hostile or non-hostile intentionality attributions	Number of respective intentionality attribution responses; RT	n/a	rTMS over RTPJ increased hostile intention interpretations and decreased non-hostile intention interpretations; rTMS over LTPJ tended to show the reverse pattern
Kelly et al., 2014	LTPJ; RTPJ	Single-pulse TMS	fMRI-guided neuronavigation	70% MSO; control (2 cm ant. to stimulation site)	Social attribution task: attribution of awareness to others (fMRI experiment); Visual detection task (subsequent TMS experiment)	Degree of awareness; PCR	n/a	single-pulse TMS over LTPJ/RTPJ associated with attribution of awareness to others disrupted subjects' attribution of awareness to themselves
Silani et al., 2013	rSMG^**^	rTMS	MRI-guided neuronavigation	1 Hz, 15 min, 110% RMT; Control (vertex); (between subj. design)	Judgments of pleasantness of self- or other-experienced visuo-tactile stimulation	Stimulation pleasantness rating	η^2^_*p*_ = 0.098	rTMS increased egocentric empathic judgments
Sowden and Catmur, 2013	RTPJ	online rTMS	MRI-guided neuronavigation	10 Hz, 6 pulses, 110% RMT; control (mid-occipital)	Imitation-inhibition task: inhibition of other-imitation;	RT; percent error	η^2^_*p*_ = 0.25	rTMS decreased imitation inhibition tendency
Young et al., 2010	RTPJ	rTMS; online rTMS (Exp.2)	fMRI-guided neuronavigation	1 Hz, 25 min, 70% MSO (Exp.2: 10 Hz, 500 ms, 60% MSO); control (5 cm post. to RTPJ)	Moral scenarios manipulating protagonists' beliefs and action outcomes	moral judgments; RT	η^2^_*p*_ = 0.50–0.56	rTMS disrupted participants' mentalizing in moral judgments, especially when actions had the goal to harm others
**MPFC**								
Enticott et al., 2011	bilateral dmPFC	rTMS	7 cm anterior to M1 along the midline	5 Hz, 30 10 s rTMS trains (20 s inter-train interval), 100% RMT; 9 sessions in 11 days	Interpersonal Reactivity Index: self-report measure of empathy; Autism Spectrum Quotient: self-report measure of autistic traits; Ritvo Autism Asperger Diagnostic Scale; Non-standardized interviews	Self-report: pre, post, 1 MO, 6 MO follow up	n/a	high-frequency rTMS lessened autistic symptoms and symptom intensity and improved social functioning in a 20-year-old woman with Asperger's syndrome
Enticott et al., 2014	bilateral dmPFC	rTMS	7 cm anterior to M1 along the midline	5 Hz, 30 10 s rTMS trains (20 s inter-train interval), 100% RMT; Sham; (between subj./double-blind design) 10 sessions every consecutive weekday	Interpersonal Reactivity Index: self-report measure of empathy; Autism Spectrum Quotient: self-report measure of autistic traits; Ritvo Autism Asperger Diagnostic Scale; Non-standardized interviews; Reading the mind in the eyes test: decoding of mental states from facial expressions; Frith-Happé-animations: mental state attribution to geometric shapes;	Self-report: pre, post, 1 MO follow up; PCR; Amount and appropriateness of mentalizing	Cohen's *d* = 0.41–1.41	high-frequency rTMS (compared to sham stimulation) reduced social relating impairments and anxiety in social situations in individuals with Asperger's syndrome or high-functioning autism; No effects on experimental tasks
Krause et al., 2012	bilateral dmPFC	rTMS	7 cm anterior to M1 along the midline	1 Hz, 15 min, 100% RMT; Sham	Yoni task: cognitive and affective mentalizing; Reading the mind in the eyes test: decoding of mental states from facial expressions	PCR; RT	Cohen's *d* = 0.66–0.81	rTMS reduced affective ToM performance in subjects with high self-reported empathy and increased affective ToM performance in subjects with low self-reported empathy
Lev-Ran et al., 2012	Ventromedial MPFC	rTMS	MRI-guided neuronavigation (based on scans from 3 subjects) to BA11,12	1 Hz, 100 stimuli before each of 4 subtests, 100% RMT (of right foot); Sham (to a superior temporal region); (between subj. design)	Yoni task: affective mentalizing, judgment of character's gaze direction	RT	n/a	rTMS affected learning mechanisms in the affective mentalizing task
Schuwerk et al., 2014b	pMPFC	rTMS	1.5 cm anterior to 1/3 of the distance from nasion to inion	1 Hz; 33 min, 100% RMT; sham	False belief task requiring the computation of another's and one's own belief	PCR; RT	η^2^_*p*_ = 0.24	rTMS impaired the ability to distinguish the other's from one's own perspective
**DLPFC/IFG**								
Berlim et al., 2012	lDLPFC	rTMS	10/20 EEG position F3	10 Hz, 75 4 s rTMS trains (26 s inter-train interval); 120% RMT; daily for 4 weeks	Reading the mind in the eyes test: decoding of mental states from facial expressions; HaM-d_21_ (Hamilton, 1960): clinician-rated measure of major depression intensity	PCR; Rating scores	n/a	Performance in Reading the mind in the eyes test improved in proportion to major depression symptoms improvement
Costa et al., 2008	lDLPFC; rDLPFC;	rTMS	Neuronavigation to a priori defined coordinates	1 Hz, 15 min, 90% RMT; sham	Short stories: false belief/faux pas/control	PCR; RT	n/a	rTMS over rDLPFC impaired performance in false belief task; rTMS over rDLPFC and lDLPFC impaired performance in faux pas test
Kalbe et al., 2010	rDLPFC	rTMS	MRI-guided neuronavigation, combined with 10/20 EEG position F4 and 5 cm-rule	1 Hz, 15 min; 100% RMT; control (vertex)	Yoni task: cognitive and affective mentalizing	PCR; RT	n/a	rTMS over rDLPFC accelerated RTs in cognitive mentalizing and had no effect on affective mentalizing
Keuken et al., 2011	lIFG	rTMS	10/20 EEG position F7	1 Hz, 5 min; 45% MSO; control (vertex, 10/20 EEG position Cz); (between subj. design)	Modification of the Reading the mind in the eyes test: decoding of mental states from facial expressions; Modification of cartoon task Brunet et al., 2000: attribution of belief and intentions; reasoning about physical causations	PCR; RT	n/a	No effect of rTMS on task performance

RTPJ, right temporoparietal junction; LTPJ, left temporoparietal junction; rTMS, repetitive transcranial magnetic stimulation; RMT, resting motor threshold; RT, reaction time; PCR, percentage of correct responses; (f)MRI, (functional) magnetic resonance imaging; MSO, maximum stimulator output; rSMG, right supramarginal gyrus; dmPFC, dorsomedial PFC; M1, primary motor cortex; BA, Brodmann area; MPFC, medial prefrontal cortex; (r/l)DLPFC (right/left) dorsolateral PFC, lIFG, left inferior frontal gyrus;

* effect size of statistical analysis/analyses which was/were central for the interpretation of TMS effects

** the rSMG as targeted in this study is anterior to the RTPJ.

## What TMS reveals about mentalizing

### The neurocognitive basis of mentalizing

**Figure 1:**
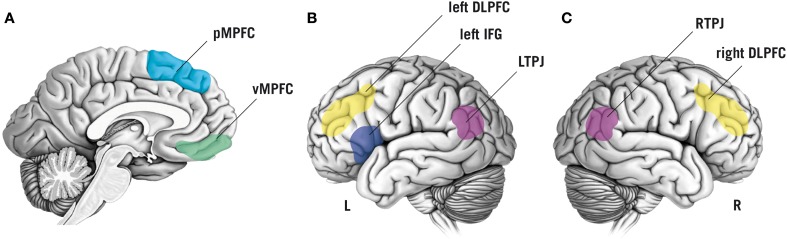
**Schematic overview of brain regions that have been targeted by non-invasive brain stimulation to study functional and dysfunctional mentalizing**. Color labels are approximate. **(A)** Sagittal view of the brain showing the posterior medial prefrontal cortex (pMPFC, indicated in light-blue) and the ventromedial prefrontal cortex (vMPFC, in green). **(B)** Lateral view of the left (L) hemisphere of the brain. Colored regions display the left inferior frontal gyrus (IFG; in dark-blue), the left dorsolateral prefrontal cortex (DLPFC; in yellow), and the left temporoparietal junction (LTPJ; in purple). **(C)** Lateral view of the right (R) hemisphere: the right DLPFC is displayed in yellow, the right temporoparietal junction (RTPJ) in purple.

Based on a large corpus of findings about the neurophysiological basis of mentalizing (e.g., Van Overwalle, 2009; Mar, 2011), neuroscientific methods are increasingly employed to test specific hypotheses about the neurocognitive processes that constitute mentalizing. Here, we focus on brain regions that appear to be central for mentalizing and have been targeted in TMS studies thus far, namely the temporoparietal junction (TPJ), the dorsolateral prefrontal cortex (DLPFC), the inferior frontal gyrus (IFG), and the medial prefrontal cortex (MPFC; Figure 1).

#### Right TPJ and mental models of self and other

Consistently observed in neuroimaging studies on mentalizing, but little understood, is functional activity of the right TPJ (RTPJ)^1^. Costa et al. (2008) were the first to show that stimulation of the RTPJ interferes with mentalizing. Applying 1 Hz-rTMS impaired subjects' performance in a false belief task (questions on stories describing false beliefs about an object's location) and in a Faux Pas Test (questions on stories describing protagonists' mental states that led to awkward behavior). Consistently, Giardina et al. (2011) and Young et al. (2010) found that 1 Hz (/10 Hz-online)-rTMS over the RTPJ influenced the use of mental states in reasoning about ethical or unethical behavior of others.

Crucially, TMS can not only tell us that the RTPJ is involved in mentalizing, it also advances our understanding of its underlying function. As outlined below, recent TMS studies provide converging evidence for the idea that RTPJ's function in mentalizing is to handle internal models of one's own and other's mental states and their relation to the environment (Decety and Sommerville, 2003).

Several lines of evidence from neuroimaging and brain stimulation in single cases suggested that the RTPJ integrates multisensory input with internally stored information to form a first-person perspective, i.e., a coherent sense of one's own body situated in and distinguishable from the rest of the world (cf., Blanke et al., 2005; De Ridder et al., 2007; Ionta et al., 2011). This notion has been supported by a study in which RTPJ activity has been disrupted by single-pulse TMS during the rubber hand illusion, resulting in an impaired ability to distinguish between self-relevant (“this is part of my body”) and self-irrelevant (“this is not part of my body”) sensory information (Tsakiris et al., 2008). It was concluded that the RTPJ maintains an internal model of one's own body as a reference for self-relevance evaluations of incoming sensory information.

Heinisch et al. (2011; cf., Heinisch et al., 2012) provided evidence that the RTPJ is also involved in distinguishing self-relevant from other-relevant information. In this study subjects were presented with a picture of another person's face that morphed gradually into a picture of their own face or vice versa. The participants indicated the moment they recognized their own face, and the moment they were sure that they were seeing the face of another person, respectively. The application of 1 Hz-rTMS over the RTPJ biased this self-other discrimination toward self-face recognition at the expense of other-face recognition. Further evidence comes from a control-of-imitation task, in which the participants moved a finger either congruently or incongruently to a simultaneously observed finger movement of another person. The ability to control the tendency to imitate the other's movement was impaired by 10-Hz-online-rTMS (Sowden and Catmur, 2013)^2^.

In sum, these findings confirm the role of the RTPJ in mentalizing and specify that the RTPJ is critically relevant for simultaneously maintaining mental models of the self and others and their relation to the environment (“how do I perceive the world vs. how does the other perceive the world?”). This may be achieved by integrating sensory information from the environment with internally stored information (Cabeza et al., 2012) and with expectations and predictions about self and other (Koster-Hale and Saxe, 2013). By this we are able to flexibly switch between perspectives, depending on what is required in a certain situation.

#### DLPFC, IFG, and perspective inhibition

Evidence on the role of the DLPFC in mentalizing is still too sparse to draw firm conclusions about its neurocognitive role in mentalizing (cf., Costa et al., 2008; Kalbe et al., 2010). Further, only little TMS research has focused on the more ventral IFG, a region with much larger evidence on its involvement in mentalizing (Mar, 2011). It has been proposed that the IFG's role in mentalizing is perspective inhibition (Ruby and Decety, 2004; Ramsey et al., 2013). For example, when adopting another's perspective, one's own perspective has to be inhibited and vice versa. While the RTPJ maintains mental models of one's own and another's perspective, the IFG inhibits one of these models during perspective selection. To our knowledge only one study investigated the effect of rTMS over the left IFG (Keuken et al., 2011). In this study a relatively short 1 Hz-rTMS of 5 min had no effect on subsequent performance in two standard mentalizing tasks as compared to control stimulation. More studies employing well-suited tasks and stimulation protocols are required to test the IFG's causal role in mentalizing.

#### MPFC and decoupling

It was proposed that the MPFC's role in mentalizing is to subserve the decoupling mechanism, i.e., processing another's perspective independently from one's own view on the world (Leslie, 1987, 1994; Frith and Frith, 2003; Gallagher and Frith, 2003; Döhnel et al., 2012). Recent neuroimaging findings showed that during the computation of one's own and another's perspective, the posterior MPFC (pMPFC) is involved in establishing a perspective difference through inhibitory influence on temporoparietal brain regions (Schuwerk et al., 2014a). It seems that while the RTPJ maintains mental models of self and other by integrating internal (memory-based/predicted) and external (sensory) information, inhibitory influence of the pMPFC suppresses processing of external information to enable the decoupled computation of one's own and another's perspective. Consistent with this idea, inhibiting the pMPFC by 1 Hz-rTMS with a double-cone coil impaired the participant's ability to distinguish between one's own and another's perspective in a false belief task (Schuwerk et al., 2014b). In another study, 1 Hz-stimulation of the pMPFC with so-called “deep rTMS” modulated affective ToM performance in dependence of baseline empathic abilities (Krause et al., 2012). Taken together, these findings indicate that the pMPFC can be targeted by specific rTMS techniques and encourage future rTMS research focusing on that area.

### TMS to study and treat dysfunctional mentalizing?

Recently, researchers began to test the therapeutic use of high-frequency rTMS on dysfunctional mentalizing in ASD and MDD. However, little is known about specifically impaired underlying neurocognitive mechanisms: ASD is characterized by widespread structural and functional brain abnormalities (Philip et al., 2012; Mueller et al., 2013). Among these, a reduced functional connectivity between the MPFC and RTPJ during mentalizing has been observed in individuals with ASD (Castelli et al., 2002; Kana et al., 2009). It can be hypothesized that the decoupled processing of one's own and another's perspective, mediated by inhibitory influence of the pMPFC to the temporoparietal cortex, is impaired in ASD. This is supported by the specific difficulty to attribute false beliefs to other people (Baron-Cohen et al., 1985; Senju et al., 2009), and close to the early hypothesis that a “failure of decoupling” underlies ASD (Leslie, 1987). If this were the case, could high-frequency stimulation of the pMPFC alleviate this mentalizing deficit?

In a double-blind randomized sham-controlled trial, Enticott et al. (2014) tested if high-frequency rTMS over the pMPFC improves impaired social functioning in ASD. Participants with high-functioning autism and Asperger's syndrome received 5 Hz-rTMS of the pMPFC on consecutive days over about 2 weeks. After active treatment as compared to sham treatment, patients reported reduced social relating impairments and anxiety in social situations (Enticott et al., 2014), as well as improved social functioning, including an increased capacity for perspective taking and empathy (Enticott et al., 2011).

Taken together, this fits with the hypothesis that the mentalizing deficit in ASD is related to impaired inhibitory influence of the pMPFC on the RTPJ. However, to provide direct evidence for this idea, future TMS studies have to show that (1) high-frequency TMS increases the connectivity between the pMPFC and RTPJ during mentalizing and (2) improves the ability to establish a perspective difference in a sensitive experimental task.

Clinical research indicates that a mentalizing deficit also plays a role in MDD (Schreiter et al., 2013). Compared to non-depressed controls, individuals with a current depressive episode showed a weaker performance in decoding mental states from facial expressions (Lee et al., 2005). Patients with a currently remitted MDD were impaired in a second-order false belief task (inferring thoughts about thoughts; Inoue et al., 2004) and had a higher risk for relapse 1 year later (Inoue et al., 2006). Also mentalizing-associated brain regions show abnormal functional activity and connectivity in depression (Aan Het Rot et al., 2009; Price and Drevets, 2012).

Is the neurocognitive basis of mentalizing affected in MDD? A prominent symptom of depressed patients is a high self-focus, i.e., a high attentional focus on oneself compared to others (e.g., Flory et al., 2000). An impaired ability to efficiently switch between one's own and another's perspective might be one contributing factor to this predominant self-focus. One's own negatively biased perspective constitutes the reference-point not only in judgments of one's own current and future situation, but also affects how one perceives the rest of the world (cf. the negative triad; Beck, 1972).

Presently, this idea remains speculative. Although a large body of evidence suggests that rTMS of the DLPFC has an antidepressant effect (Lefaucheur et al., 2014), we currently lack evidence on possible links between impaired neurocognitive components of mentalizing and depressive symptoms. To our knowledge, only one study has addressed this issue and found a relation between 10 Hz-rTMS to the left DLPFC, improved performance in a ToM task, and the alleviation of depressive symptoms (Berlim et al., 2012). However, these preliminary findings do not allow for firm conclusions about causal associations of these factors.

In sum, TMS seems to be a valuable tool in the investigation of the relationship between impaired mentalizing, its neuronal correlates, and related psychiatric disorders. At the same time it is definitely premature to claim that TMS constitutes a therapeutic strategy to improve impaired mentalizing. But in the light of accumulating evidence that brain stimulation may enhance (1) cognitive functioning in psychiatric disorders (Demirtas-Tatlidede et al., 2013) and (2) mentalizing in healthy subjects (Santiesteban et al., 2012), it can be regarded as a promising method to tackle dysfunctional mentalizing.

## Effectiveness of employed stimulation methods

All previously reported stimulation techniques (online and offline rTMS, single-pulse TMS), brain site localization procedures, and most stimulation protocols produced effects of interest (Table 1). Unfortunately, only half of the TMS studies on mentalizing reviewed here reported effect sizes. In these studies the effect sizes are medium to large. To improve the evaluation of observed findings and facilitate the design of future studies, comprehensive descriptions of all methodological aspects and detailed reporting of results, including effect sizes, are highly desirable.

A critical issue appears to be the employment of sensitive experimental paradigms. Particularly, adaptations of traditional ToM tests, such as the Reading the Mind in the Eyes Test (Baron-Cohen et al., 2001), ToM cartoons (Brunet et al., 2000) or social animations (e.g., Abell et al., 2000), produced heterogeneous results. Several authors stated that their employed tasks might not be sensitive enough to measure TMS-induced effects on behavioral outcome measures (e.g., Krause et al., 2012; Enticott et al., 2014; c.f., Keuken et al., 2011; Lev-Ran et al., 2012). Future studies must carefully design paradigms that allow for detecting TMS effects on reaction times and accuracy rates, the two most prominent outcome measures.

The development of sensitive mentalizing tasks for TMS research will also be critical for the evaluation of rTMS as a therapeutic approach for dysfunctional mentalizing. In a review on the role of social cognition in MDD, Schreiter et al. (2013) pointed out that especially objective measures, i.e., laboratory tasks, seem to be more reliable and sensitive than self-reports, for example.

## Future directions and conclusions

To date, available TMS studies on mentalizing show that the RTPJ, DLPFC, and MPFC, identified by previous neuroimaging research on ToM, are causally involved in mentalizing. Given the correlational nature of functional magnetic resonance imaging or electroencephalography, this is a critical finding that adds to neuropsychological evidence on the causal role of those brain regions in ToM (e.g., Samson and Michel, 2013). Further, TMS is particularly suited to specify the neurocognitive building blocks of mentalizing, a current issue in ToM research. A major challenge for future research will be to develop sensitive paradigms to detect TMS-induced effects on mentalizing.

Future research should focus on the connectivity between mentalizing-associated brain regions. Both brain functions and stimulation effects are not restricted to specific regions, but have to be conceptualized as network effects. Only if we understand the brain as a network can we learn more about how the interplay of its regions underpins functional and dysfunctional social cognition (e.g., Kennedy and Adolphs, 2012). Although a set of brain regions which were linked to mentalizing by previous research is labeled “ToM network,” little is known about the critical interactions of those regions. One avenue for future research will be the simultaneous stimulation of several brain regions associated with mentalizing while another approach is the combination of brain stimulation and neuroimaging for the assessment of stimulation-induced network effects.

Claiming that TMS can be used as a therapeutic intervention for dysfunctional mentalizing is clearly premature. But, preliminary evidence for its influence on mentalizing in ASD and MDD promises that TMS, combined with sensitive paradigms, will provide insights into the dysfunction of the neurocognitive basis of mentalizing in clinical conditions. In the future, it may be possible to combine TMS with psychotherapeutic interventions in order to tackle impaired social cognition in psychiatric disorders.

### Conflict of interest statement

The authors declare that the research was conducted in the absence of any commercial or financial relationships that could be construed as a potential conflict of interest.

## Footnotes

^1^Also the left TPJ is involved in mentalizing (e.g., Samson et al., 2004; Schurz et al., 2014). However, because recent theoretical discussions focus on the RTPJ and the TMS studies reviewed here also predominantly targeted the RTPJ, we concentrate on this region in the current article.

^2^A similar effect was previously shown by Santiesteban et al. (2012) using the same task but a different stimulation method, namely transcranial direct current stimulation. Intriguingly, anodal tDCS (which increases cortical excitability) enhanced the ability to control imitation. Moreover, Santiesteban and colleagues showed that anodal tDCS not only facilitated the self at the expense of the other, but also vice versa. In another task, anodal stimulation facilitated the subjects' ability to inhibit their own perspective in order to adopt the perspective of another person. We point at this finding because of its high theoretical relevance. However, it is difficult to directly compare tDCS and rTMS findings as those two methods substantially differ. We refrain from a detailed discussion of this tDCS study as this falls beyond the scope of this review. For a general overview of both noninvasive brain stimulation methods see Wagner et al. (2007).
